# Temperature variability and mortality risk: distinguishing intraday and interday effects and quantifying the attributable mortality burden in Chengdu, Southwest China

**DOI:** 10.3389/fpubh.2026.1809630

**Published:** 2026-05-29

**Authors:** Jiaqi Huang, Dan Kuang, Jingwen Sun, Jinqiu Yao, Mingming Han, Nan Du, Fangkui Qin, Yifan Zhai, Yueling Li, Wei Huang, Cheng Wang, Rong Lu, Xufang Gao

**Affiliations:** Chengdu Center for Disease Control and Prevention (Chengdu Institute of Health Supervision), Chengdu, Sichuan, China

**Keywords:** interday, intraday, mortality, mortality burden, temperature variability

## Abstract

**Background:**

With the ongoing global climate change, the adverse health impacts of extreme weather and climate events such as heatwaves and cold spells have been widely investigated, while the effects of temperature variability (TV) remain insufficiently explored, and the differential impacts of intraday TV and interday TV are largely unidentified.

**Objectives:**

To investigate the associations between three TV indices (total TV, intraday TV, interday TV) and mortality, and to quantify the TV-attributable mortality burden in Chengdu.

**Methods:**

Daily meteorological and mortality data in Chengdu from 2015 to 2024 were collected. The three TV indices were derived from different forms of standard deviations of daily minimum/maximum/mean temperatures. Distributed lag non-linear models (DLNMs) were used to assess the associations between TV indices and mortality. Comprehensive subgroup analyses stratified by cause of death, gender, age group, and season were conducted to identify vulnerable populations. The mortality burden attributable to TV was quantified using attributable numbers of deaths (AN) and attributable fractions (AF).

**Results:**

Total TV and intraday TV were significantly positively associated with daily mortality in Chengdu, while interday TV showed a negative association. For each interquartile range (IQR) increase in total TV, intraday TV and interday TV, the corresponding all-cause mortality risk increased by 1.4% (0.5, 2.3%), 2.9% (2.0, 3.8%), and −1.7% (−2.3, −1.1%), respectively. The AN of all-cause deaths attributable to intraday TV was 27,186 (18,527, 35,767), and the AFs of intraday TV for all-cause, cardiovascular disease-related, and respiratory disease-related deaths were 2.82% (1.92, 3.71%), 2.83% (1.68, 3.97%), 5.19% (3.80, 6.56%), respectively. Subgroup analyses identified deaths due to respiratory diseases, females, and individuals aged ≥65 years as vulnerable populations. Additionally, the adverse effect of intraday TV was more pronounced during the cold seasons.

**Conclusion:**

This study suggested that short-term temperature variations elevate the mortality risk in Chengdu, with intraday TV as the primary driver of the increased mortality risk. These findings enrich evidence on TV-related health impacts, highlight the need to distinguish intraday and interday TV in research and practice, and support local climate-health adaptation and intervention strategies.

## 1 Introduction

Climate change is one of the most critical challenges confronting the world today (1). Notably, the Intergovernmental Panel on Climate Change (IPCC) highlights that human activities have driven a 1.1 °C increase in global surface temperatures over the past century; the adverse impacts and risks associated with climate change are projected to intensify in the forthcoming decades (2). Numerous studies have shown that both low and high temperatures are linked to an increased risk of mortality (3–6). With ongoing global warming, extreme weather and climate events, such as heatwaves and cold spells, are increasing in frequency and intensity, posing severe threats to human health and socioeconomic systems (7). Beyond extreme temperature events, climate change is also driving increased temperature variations (8). However, the impacts of temperature variations on human health remain inconclusive in the existing literature (9). Although humans have demonstrated a certain degree of adaptability to gradual rises in temperature as climate change progresses, their capacity to cope with short-term temperature fluctuations remains largely limited (8, 10). Consequently, there is a critical need to explore the potential impacts of temperature variations on health.

Temperature variability indices refer to a set of indicators that characterize the magnitude of temperature variation within a specific short-time window, including the diurnal temperature range (DTR), temperature changes between neighboring days (TCN), and temperature variability (TV) (8, 11, 12). However, conventional indices, including DTR and TCN, only capture absolute temperature changes without accounting for potential lag effects, thereby limiting their ability to characterize the continuous and dynamic nature of temperature fluctuations (8, 10). The composite TV index (total TV), proposed by Guo et al. (8), reflects temperature variation within a specific exposure period by calculating the standard deviation of daily maximum and minimum temperatures during the given period. It characterizes temperature variation more comprehensively and accurately, and has been consistently associated with a range of adverse health outcomes (13–17).

Nonetheless, total TV fails to distinguish the separate effects of intraday and interday temperature variation (10). From physiological and behavioral perspectives, humans differ fundamentally in their responses to intraday and interday temperature fluctuations. Interday fluctuations can be anticipated via weather forecasts, enabling timely protective behaviors. In contrast, the sudden and unpredictable character of intraday fluctuations impedes rapid thermoregulatory adaptation, thereby elevating the risks of adverse health outcomes (10, 18). Thus, Wen et al. further decomposed total TV into intraday and interday components to explore their independent and separate health effects, which has further clarified the characteristics of temperature variations along different dimensions (18). However, no studies have yet investigated the impacts of temperature variations on mortality in Southwest China, nor explored whether these impacts are driven by its intraday or interday components.

Located in the western Sichuan Basin, Chengdu is the capital of Sichuan Province and a key economic center in southwestern China. Affected by the topographic barrier of the basin and its subtropical humid climate, temperature variations in Chengdu exhibit unique regularity and regional specificity (19, 20). As an officially designated megacity in China with a permanent resident population exceeding 21.47 million, Chengdu has a prominent aging population and a significant mortality burden. Therefore, revealing the association between temperature variations and mortality in Chengdu is of great regional representativeness and public health significance.

Based on 10-year time-series of continuous surveillance data on meteorology and mortality, this study aims to address the underexplored aspects of the health impacts of temperature variations in the context of an inland subtropical urban areas in Southwest China. Specifically, we will first investigate the association between total TV and mortality, with a focus on distinguishing the independent effects of intraday TV and interday TV. Then, to quantify the TV-related mortality burden, we will further calculate the corresponding attributable numbers of deaths (AN) and attributable fractions (AF) for the three TV indices (total TV, intraday TV, and interday TV). Furthermore, comprehensive subgroup analyses will be conducted by cause of death, gender, age group, and season to identify vulnerable populations susceptible to short-term temperature fluctuations. Collectively, the findings of this study are expected to inform targeted evidence for optimizing public health strategies to mitigate temperature variations-induced mortality risks in Chengdu.

## 2 Materials and methods

### 2.1 Data collection

We collected daily meteorological and mortality data in Chengdu from January 1, 2015, to December 31, 2024. The monitoring data of meteorological factors, including daily mean temperature (°C), daily maximum temperature (°C), daily minimum temperature (°C) and relative humidity (%), were acquired from the Chengdu Meteorological Office. Daily mortality data were obtained from the China’s National Information System for Disease Control and Prevention, and systematically categorized into four clinically defined groups according to the International Classification of Diseases, 10th Revision (ICD-10): all-cause deaths, non-accidental deaths, respiratory disease-related deaths (ICD-10: J00-J99), and cardiovascular disease-related deaths (ICD-10: I00-I99). We further organized the mortality data by cause-specific, gender-specific (male, female), age group-specific (0–15 years, 16–64 years, ≥65 years), and season-specific (cold seasons: November–April, warm seasons: May–October) categories.

### 2.2 Temperature variability indices

This study defined the temperature variability in Chengdu using three indices: namely total TV, intraday TV, and interday TV. Proposed by Guo et al. (8), total TV was defined as the standard deviation (SD) of daily minimum and maximum temperatures within a time window of *L* days preceding the current day, and could be calculated by the following equation


$$\text{Total}\;TV_{0-L}=\sqrt{\frac{\sum {\left(T_{\text{minl}}-\bar{T}\right)}^{2}+\sum {\left(T_{\text{maxl}}-\bar{T}\right)}^{2}}{2L+1}}$$

where *L* represents the number of preceding days used to define the lag effect. $\mathrm{Tmi}n_{l}$ and $\mathrm{Tma}x_{l}$ stand for the daily minimum and maximum temperatures on day *l*, respectively. $\bar{T}$ denotes the average temperature calculated from daily minimum and maximum temperatures across the *L + 1* days.

As suggested by Wen et al., total TV was then decomposed into two statistically independent and conceptually distinct components: the intraday TV (quantifying within-day temperature variations) and interday TV (characterizing between-day temperature variations) (18). Intraday TV was calculated as the weighted average SD of daily minimum and maximum temperatures on each day within the *L + 1* lag days, while interday TV was computed as the weighted SD of daily mean temperature during the *L + 1* lag days (18). The details are as follows,


$$\text{Intraday}\;TV_{0-L}=\sqrt{\frac{\sum {\left(T_{\text{minl}}-{\bar{T}}_{l}\right)}^{2}+\sum {\left(T_{\text{maxl}}-{\bar{T}}_{l}\right)}^{2}}{2L+1}}$$

$$\text{Interday}\;TV_{0-L}=\sqrt{\frac{2\times \sum {\left({\bar{T}}_{l}-\bar{T}\right)}^{2}}{2L+1}}$$

where $\bar{T_{l}}$ denotes the average temperature of daily minimum and maximum temperature on day *l*, which can be approximated by the daily mean temperature according to the recommendation of the World Meteorological Organization (WMO) (18, 21). The relationships among the three TV indices are as follows (18).


$$\text{Total}\;TV_{0-L^{2}}=\text{Intraday}\;TV_{0-L^{2}}+\text{Interday}\;TV_{0-L^{2}}$$

Based on the orthogonal variance decomposition, $\text{Intraday}\;TV_{0-L}$ and $\text{Interday}\;TV_{0-L}$ are statistically independent, with the squared $\text{Total}\;TV_{0-L}$ equal to the sum of the squared $\text{Intraday}\;TV_{0-L}$ and squared $\text{Interday}\;TV_{0-L}\;$ (18).

### 2.3 Statistical analysis

#### 2.3.1 Distributed lag non-linear model

To explore the associations between temperature variation and mortality, we constructed two time-series models using a standard quasi-Poisson regression framework with key specifications guided by previous research (8, 10, 18). Consistent with our preliminary analysis as well as existing evidence demonstrating linear associations between the three TV indices and mortality, all three TV indices were incorporated into the models as linear terms: the first model included total TV alone in linear form, while the second model simultaneously incorporated intraday TV and interday TV, both specified as linear terms; additionally, a lag interval of 0–7 days was selected for all three TV indices to capture their short-term lagged effects, in line with prior studies (8, 18, 22, 23). Specifically, the models were constructed as follows,


$$Y_{t}∼\text{Quasi}-\text{Possion}(\mu_{t})$$

$$\begin{array}{l} \log(\mu_{t})=\alpha +\text{βTVindice}s_{0-L}+\mathrm{CO}V_{s}\\ +\mathrm{ns}(\text{time},\mathrm{df})+\mathrm{do}w_{t}+\text{holida}y_{t} \end{array}$$

where $Y_{t}$ represents the daily reported death count on day *t*, which is assumed to follow a quasi-Poisson distribution with $\mu_{t}\equiv E(Y_{t})$ . α is the intercept. $\text{TVindice}s_{0-L}$ stand for $\text{Total}\;TV_{0-L}$ , $\text{Intraday}\;TV_{0-L}$ and $\text{Interday}\;TV_{0-L}$ . β denotes the coefficient corresponding to the exposure-response association. COVs refer to the variables adjusted for in the model, including daily mean temperature and relative humidity. Distributed lag non-linear models (DLNMs) were applied to simultaneously capture the non-linear and lagged effects of these two covariates (24, 25). Based on prior studies (8, 10, 18, 26), natural splines (*ns*) with 4 degrees of freedom (df) were specified for both the exposure-response and lag-response dimensions of daily mean temperature and relative humidity. In the DLNM cross-basis function, a maximum lag of 21 days was specified, with equally spaced knots on the log scale of lag days. Additionally, the *ns* with 7 df per year were used to control the long-term trends and seasonal fluctuation, and $\mathrm{do}w_{t}$ and $\text{holida}y_{t}$ were dummy variables representing day-of-the-week and holiday effects, respectively.

The associations between the three TV indices and mortality were characterized by the percentage change (%) in mortality risk corresponding to an interquartile range (IQR) increase in each index, with a 95% confidence interval (95% CI) presented for each estimate (8, 10, 18).

#### 2.3.2 Attributable burden

To assess the mortality burden attributable to temperature variations, we further calculated the attributable number of deaths (AN) and the corresponding attributable fractions (AF) using the following formulas (10, 18, 22),


$$RR_{t}=\exp(\beta \times \text{TVindice}s_{0-L})$$

$$AN_{t}=\text{Number of deaths}\times \frac{RR_{t}-1}{RR_{t}}$$

$$\text{AN}=\frac{\sum AN_{t}}{\text{Total number of deaths}}$$

where β is the estimated association coefficient between the corresponding $\text{TVindice}s_{0-L}$ and mortality. $\text{Number of}\;\text{death}s_{t}$ stands for the average daily death counts over the *0-L* days lag period, while Total number of deaths denotes the total number of deaths during the whole study period.

### 2.4 Subgroup analysis

Based on mortality data stratified by cause of death, gender, age group, and season, we further conducted subgroup analyses to identify the characteristics of potentially vulnerable populations.

### 2.5 Sensitivity analysis

To verify the robustness of the findings, we conducted sensitivity analyses on key parameter selections, including the lag intervals of the three TV indices (lag 0–1 to 0–7 days), the df of the *ns* function (from 3 to 6) and the maximum lag days (21–28 days) for daily mean temperature and relative humidity. In addition, we further explored whether the COVID-19 pandemic exerted potential impacts on the results.

All analyses were conducted using R software (version 4.4.1). The significance level was set at α = 0.05.

## 3 Results

### 3.1 Descriptive statistics

From 2015 to 2024, a total of more than 960 thousand all-cause deaths were reported in Chengdu, with a daily mean of 264 deaths. Deaths attributable to cardiovascular and respiratory disease accounted for 30.31 and 23.02% of total deaths, respectively. The male-to-female ratio was 1.43, and 78.96% of all deaths occurred among individuals aged 65 years and older. The daily mean temperature was 17.07 °C. The IQRs for total TV, intraday TV, and interday TV were 1.70, 1.61, and 0.81, respectively. A detailed summary of the variables is provided in Table 1.

**Table 1:** Descriptive analysis of daily mortality, meteorological factors, and the three TV indices in Chengdu, 2015–2024.

Variables	Mean	SD	Median	*IQR* (*P_25_*, *P_75_*)	Total
All-cause deaths	264	105	249	(221, 285)	965,284
Cardiovascular disease-related deaths	80	34	75	(63, 89)	292,618
Respiratory disease-related deaths	61	50	52	(43, 67)	222,213
Male deaths	155	64	147	(130, 167)	567,328
Female deaths	109	43	102	(90, 120)	397,956
Deaths aged 0–15 years	2	2	2	(1, 3)	7,612
Deaths aged 16–64 years	54	10	53	(47, 59)	195,490
Deaths aged ≥65 years	209	98	195	(168, 227)	762,182
Daily mean temperature (°C)	17.07	7.47	17.50	(10.25, 23.60)	–
Daily maximum temperature (°C)	21.65	8.15	22.10	(14.90, 28.60)	–
Daily minimum temperature (°C)	13.56	7.34	14.40	(7.40, 20.00)	–
Relative humidity (%)	78.87	9.34	79.50	(73.00, 85.50)	–
Total TV	4.79	1.23	4.74	(3.88, 5.58)	–
Intraday TV	4.29	1.17	4.25	(3.44, 5.05)	–
Interday TV	1.41	0.66	1.27	(0.93, 1.74)	–

### 3.2 Associations among the three TV indices and mortality

Pearson correlation coefficients among the three TV indices and daily mean temperature are shown in Table 2. Specifically, total TV exhibited a strong correlation with intraday TV (*r* = 0.970), whereas it showed a moderate correlation with interday TV (*r* = 0.427). Correlation coefficients for other lag intervals (from 0–1 to 0–6 days) are reported in Supplementary material.

**Table 2:** Pearson correlation coefficients among the three TV indices and daily mean temperature.

Variables	Tmean	Total TV	Intraday TV	Interday TV
Tmean	1.000			
Total TV	0.237	1.000		
Intraday TV	0.285	0.970	1.000	
Interday TV	−0.067	0.427	0.221	1.000

Tmean denotes daily mean temperature.

**Figure 1:**
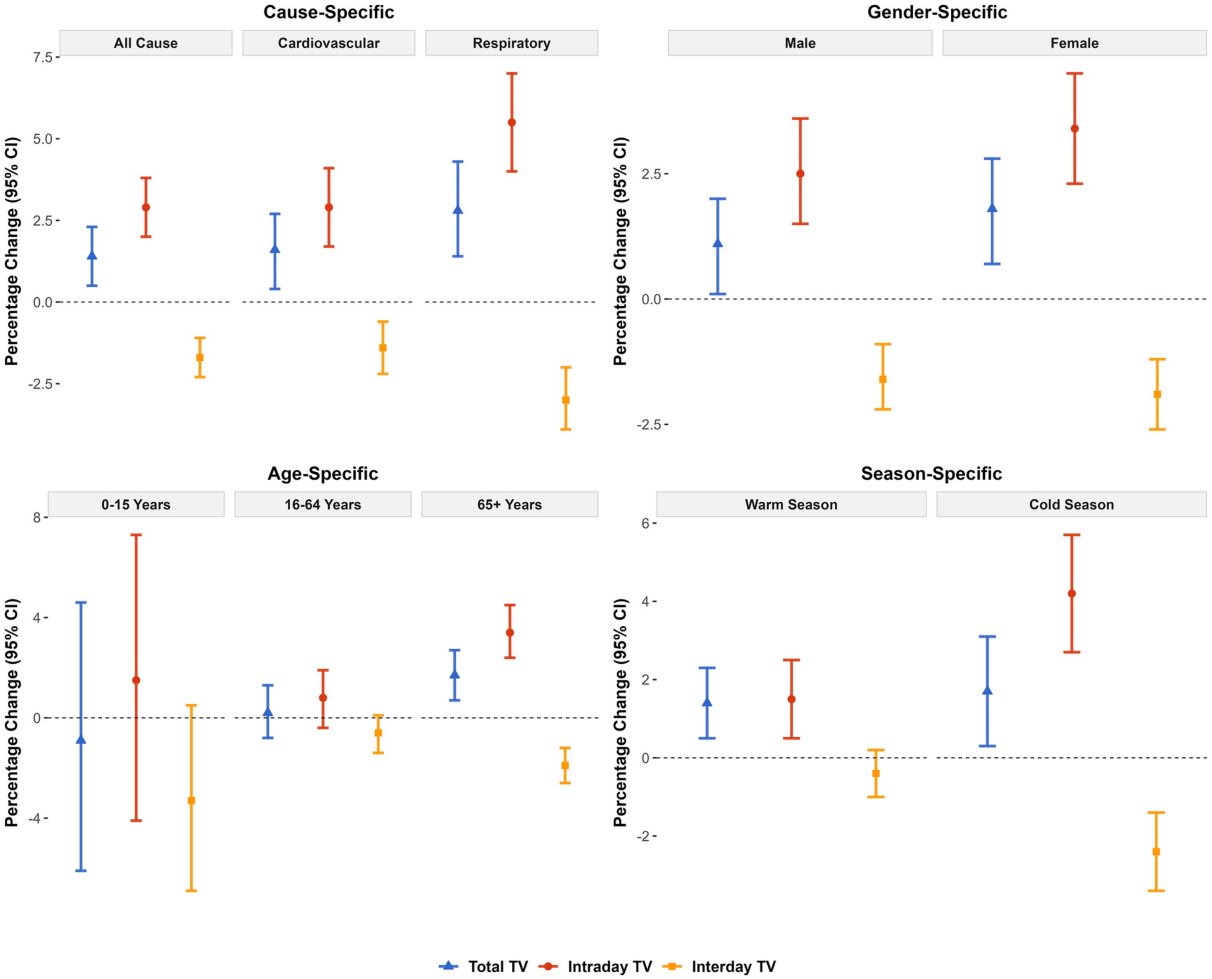
Percentage change in mortality risk associated with each IQR increase in the three TV indices across various subgroups.

Figure 1 presents the percentage change in mortality risk associated with each IQR increase in the three TV indices across the cause-, gender-, age-, and season-stratified subgroups. The exact values are provided in Table 3. Among the three TV indices, total TV and intraday TV both had a significant positive association with all-cause deaths, with respective percentage changes in mortality risk of 1.4% (0.5, 2.3%) and 2.9% (2.0, 3.8%) per IQR increase, while interday TV showed a significant negative association at −1.7% (−2.3, −1.1%). Subgroup analyses revealed that intraday TV showed stronger associations with mortality risk in individuals with respiratory diseases, females, those aged 65 years and older, and during the cold season.

**Table 3:** Percentage changes in mortality risk associated with each IQR increase in the three TV indices.

Variables	Total TV	Intraday TV	Interday TV
All-cause deaths	1.4 (0.5, 2.3)	2.9 (2.0, 3.8)	−1.7 (−2.3, −1.1)
Cardiovascular disease-related deaths	1.6 (0.4, 2.7)	2.9 (1.7, 4.1)	−1.4 (−2.2, −0.6)
Respiratory disease-related deaths	2.8 (1.4, 4.3)	5.5 (4.0, 7.0)	−3.0 (−3.9, −2.0)
Male deaths	1.1 (0.1, 2.0)	2.5 (1.5, 3.6)	−1.6 (−2.2, −0.9)
Female deaths	1.8 (0.7, 2.8)	3.4 (2.3, 4.5)	−1.9 (−2.6, −1.2)
Deaths aged 0–15 years	−0.9 (−6.1, 4.6)	1.5 (−4.1, 7.3)	−3.3 (−6.9, 0.5)
Deaths aged 16–64 years	0.2 (−0.8, 1.3)	0.8 (−0.4, 1.9)	−0.6 (−1.4, 0.1)
Deaths aged ≥65 years	1.7 (0.7, 2.7)	3.4 (2.4, 4.5)	−1.9 (−2.6, −1.2)
Warm season deaths	1.4 (0.5, 2.3)	1.5 (0.5, 2.5)	−0.4 (−1.0, 0.2)
Cold season deaths	1.7 (0.3, 3.1)	4.2 (2.7, 5.7)	−2.4 (−3.4, −1.4)

### 3.3 Attributable burden

Table 4 shows the mortality burden attributable to each of the three TV indices. For all-cause deaths, total TV and intraday TV showed positive attributable burdens, with corresponding ANs of 12,889 (4,417, 21,287) and 27,186 (18,527, 35,767), and AFs of 1.34% (0.46, 2.21%) and 2.82% (1.92, 3.71%), respectively. By contrast, interday TV exhibited a negative attributable burden, with an AN of −16,815 (−22,876, −10,790) and an AF of −1.74% (−2.37, −1.12%). Specifically, intraday TV was associated with the highest AFs across all subgroups.

**Table 4:** Attributable numbers of deaths (AN) and attributable fractions (AF) for the three TV indices.

Variables	Total TV	Intraday TV	Interday TV
All-cause deaths			
AN	12,889 (4,417, 21,287)	27,186 (18,527, 35,767)	−16,815 (−22,876, −10,790)
AF (%)	1.34 (0.46, 2.21)	2.82 (1.92, 3.71)	−1.74 (−2.37, −1.12)
Cardiovascular disease-related deaths			
AN	4,492 (1,214, 7,734)	8,285 (4,908, 11,623)	−4,119 (−6,466, −1790)
AF (%)	1.54 (0.41, 2.64)	2.83 (1.68, 3.97)	−1.41 (−2.21, −0.61)
Respiratory disease-related deaths			
AN	6,070 (3,005, 9,092)	11,529 (8,446, 14,568)	−6,800 (−9,102, −4,521)
AF (%)	2.73 (1.35, 4.09)	5.19 (3.80, 6.56)	−3.06 (−4.10, −2.03)
Male deaths			
AN	5,958 (573, 11,292)	14,078 (8,560, 19,541)	−9,170 (−13,015, −5,350)
AF (%)	1.05 (0.10, 1.99)	2.48 (1.51, 3.44)	−1.62 (−2.29, −0.94)
Female deaths			
AN	6,928 (2,846, 10,967)	13,109 (8,937, 17,237)	−7,653 (−10,591, −4,735)
AF (%)	1.74 (0.72, 2.76)	3.29 (2.25, 4.33)	−1.92 (−2.66, −1.19)
Deaths aged 0–15 years			
AN	−68 (−493, 334)	110 (−322, 518)	−257 (−564, 39)
AF (%)	−0.90 (−6.48, 4.39)	1.44 (−4.23, 6.81)	−3.37 (−7.41, 0.51)
Deaths aged 16–64 years			
AN	470 (−1,646, 2,563)	1,491 (−706, 3,664)	−1,205 (−2,696, 275)
AF (%)	0.24 (−0.84, 1.31)	0.76 (−0.36, 1.87)	−0.62 (−1.38, 0.14)
Deaths aged ≥65 years			
AN	12,492 (5,161, 19,751)	25,139 (17,663, 32,541)	−14,822 (−20,090, −9,590)
AF (%)	1.64 (0.68, 2.59)	3.30 (2.32, 4.27)	−1.94 (−2.64, −1.26)
Warm season deaths			
AN	10,603 (3,683, 17,460)	11,338 (4,061, 18,545)	−3,193 (−7,682, 1,270)
AF (%)	1.39 (0.48, 2.29)	1.49 (0.53, 2.43)	−0.42 (−1.01, 0.17)
Cold season deaths			
AN	12,800 (2,478, 22,982)	30,830 (20,103, 41,402)	−18,905 (−27,113, −10,782)
AF (%)	1.68 (0.33, 3.02)	4.04 (2.64, 5.43)	−2.48 (−3.56, −1.41)

### 3.4 Sensitivity analysis

Sensitivity analysis showed that the results remained robust after adjusting for key model parameters. After excluding the period with the highest COVID-19 incidence for reanalysis, the association between the three TV indices and all-cause mortality was still generally consistent with the main analysis, further verifying the reliability of our study findings. Detailed results of the above sensitivity analyses are presented in the Supplementary material.

## 4 Discussion

This is the first study in Chengdu, a southwestern Chinese provincial capital, to systematically explore the associations between three key temperature variability indices and mortality, which not only investigated the impacts of total TV but also further decomposed it into intraday and interday TV components for concurrent analysis to evaluate their independent and differential effects on mortality. Comprehensive subgroup analyses were further conducted by cause of death, gender, age group, and season. The core findings demonstrated that short-term temperature variations elevate local mortality risk, with intraday TV as the primary driver of the increased mortality risk. Deaths due to respiratory diseases, females, and individuals aged ≥65 years were identified as the vulnerable populations to short-term temperature variations, with the adverse effects being more prominent during the cold season. By disentangling the distinct roles of intraday and interday TV, our findings provide solid scientific evidence for clarifying the health impacts of temperature variations, particularly for informing targeted public health interventions against adverse health effects of temperature variations.

Currently, the indices used to characterize temperature variations in previous studies have not yet been standardized, and can be mainly categorized into three types: the diurnal temperature range (DTR), defined as the difference between the daily maximum and minimum temperatures within a single day, only quantifies the magnitude of intraday temperature fluctuations (27, 28); the temperature change between neighboring days (TCN), calculated as the absolute value of the difference in mean temperatures between two consecutive days, merely reflects interday temperature variations (12, 29); and the temperature variability (total TV), calculated as the standard deviation of daily maximum and minimum temperatures over a specified exposure period, captures both intraday and interday temperature variations simultaneously, thus providing a more comprehensive measure of temperature fluctuations over an extended time interval (8). On the basis of total TV, Wen et al. further proposed two novel sub-indices, namely intraday TV and interday TV, and verified that these two sub-indices can replace the previously used indices (DTR and TCN) to comprehensively assess the health impacts of temperature variations (18).

Our study found that elevated levels of total TV were significantly associated with increased risks of all-cause, respiratory disease-related, and cardiovascular disease-related mortality, a finding which is consistently supported by global and regional studies (4, 8, 30, 31). Potential physiological mechanisms primarily involve disruptions to cardiovascular and respiratory homeostasis, including impaired blood pressure regulation, vascular stability, and airway mucosal function (17, 32–35). Vencloviene et al. reported a positive correlation between total TV and blood pressure fluctuations (36), and Ni et al. verified that total TV raises myocardial infarction hospitalization risk, a progression driven by acute physiological changes such as enhanced platelet aggregation and vasoconstriction (37). Similarly, Zhan et al. found that total TV is an independent risk factor for the incidence of chronic obstructive pulmonary disease, providing evidence that total TV could impair respiratory mucosal function and exacerbate respiratory disease progression (38). These physiological and biochemical perturbations do not remain static; instead, they can progressively impair critical organ systems, most notably the cardiovascular and respiratory systems, whose role in sustaining blood flow and gas exchange is vital to survival, with any impairment here elevating mortality risk (17, 39, 40).

By decomposing total TV into intraday and interday components, our study identified that intraday TV is the primary driver of the increased mortality risk. The finding aligns with the results of two studies conducted by Wen et al. (18, 26). The France-based study showed that the intraday component had an AF of 1.81% (0.64, 2.97%) for all-cause mortality, more than twice that of the interday component, thus dominating the local mortality burden (18). In another study covering 758 sites across 47 countries worldwide, intraday and interday TV yields AFs of 1.45 and 0.35% for all-cause mortality, respectively, further validating the predominant contribution of the intraday component (26). From a physiological perspective, dramatic intraday temperature fluctuations make it difficult for individuals with prolonged outdoor exposure to adjust their clothing promptly, which significantly elevates the burden on the cardiovascular and respiratory systems, potentially serving as a key contributor to elevated mortality risk (32, 34, 35). However, attention should be paid to the heterogeneity of research findings: Pehlivan et al. reached an opposite conclusion in their study conducted in South Korea, suggesting that interday TV plays a dominant role in increasing mortality risk (10). The disparity in the impacts of intraday versus interday TV may stem from the varying responsiveness of thermoregulatory process (18). The human body may struggle to cope with dramatic temperature fluctuations within a single day. Such abrupt changes trigger acute thermoregulatory and cardiovascular stress and activate the sympathetic nervous system, which may increase mortality risk (41). In contrast, both animal and human studies have demonstrated that sustained heat exposure over several consecutive days can induce short-term heat acclimatization, which may improve tolerance to temperature fluctuations in consecutive days (42, 43). The observed inverse association between interday TV and mortality, which differs from some existing evidence, could be explained by two factors (10, 18, 26). First, inherent differences exist in adaptive capacity to temperature fluctuations among populations across various climatic zones and socioeconomic regions (8). Residents in Chengdu may have gradually adapted to the local climate, thereby improving physiological tolerance and alleviating the potential adverse effects of interday temperature fluctuations. Second, with enhanced public awareness of the health risks associated with temperature variations, individuals can proactively mitigate the adverse impacts of interday TV by checking weather forecasts, adjusting their clothing, and rescheduling outdoor activities in a timely manner (44). These adaptive behaviors may contribute to the observed association between interday TV and mortality. Future studies across diverse regions are warranted to clarify the associations between interday TV and mortality as well as the underlying biological mechanisms.

We quantitatively measured the mortality burden attributable to total TV, intraday TV, and interday TV in Chengdu by calculating the corresponding ANs and AFs. The results showed that both total TV and intraday TV yielded positive ANs and AFs, with intraday TV imposing a more pronounced mortality burden. This finding indicates that short-term intraday temperature variations exert a more significant impact on population health. In contrast, interday TV was associated with negative ANs and AFs, suggesting that interday TV may not be a risk factor for increased mortality. Our results are consistent with previous studies highlighting the dominant role of intraday TV in mortality burden driven by temperature variations (18, 26, 45), but they contradict a study conducted in South Korea that identified interday TV as a more important contributor (10). This discrepancy may be due to differences in regional climatic characteristics and population adaptability and vulnerability, which requires further research to confirm. In conclusion, when formulating targeted public health interventions in Chengdu, priority should be given to mitigating the adverse effects of intraday temperature variations.

Subgroup analysis demonstrated significant population and seasonal heterogeneity in the impact of temperature variations on mortality, which is consistent with several prior studies (17, 46, 47). Deaths due to respiratory diseases, females, and individuals aged ≥65 years were identified as vulnerable populations susceptible to short-term temperature fluctuations. The underlying reasons were that vulnerable populations typically exhibit impaired physiological functions, declined thermoregulatory capacity, and poor tolerance to abrupt temperature fluctuations, which may lead to their increased vulnerability to the adverse effects of temperature variations (40, 48, 49). Seasonal analysis showed that the associations between intraday TV and mortality were stronger in the cold season than in the warm season, with a higher attributable burden. This may be explained by more intense temperature fluctuations during cold seasons: the thermoregulatory burden is already substantially elevated under cold conditions, and abrupt fluctuations further trigger acute physiological responses (e.g., peripheral vasoconstriction, raised vascular resistance, increased heart rate and blood pressure, as well as elevated blood viscosity and enhanced platelet aggregation) (50, 51). Poorer physiological adaptability and greater vulnerability of susceptible populations jointly contribute to the higher mortality risk during cold periods. Therefore, health protection and intervention strategies for temperature variations should integrate population targeting and temporal specificity. On one hand, targeted interventions should be implemented for vulnerable populations, guiding them to cope appropriately with temperature fluctuations by monitoring weather forecasts and early warnings, adjusting clothing promptly, and rationally using temperature-control devices (e.g., air conditioners and heaters). On the other hand, the cold season should be designated as a critical period, with enhanced monitoring and early warning of temperature variations, as well as proactive allocation of medical resources, to reduce associated mortality risks.

Given that this study included daily mortality data from 2015 to 2024, excess deaths during the COVID-19 pandemic may have introduced bias into our results (52). Therefore, we excluded the COVID-19 peak period in our sensitivity analysis. The findings were generally consistent with those of the main analysis, indicating that the observed associations between the three TV indices and mortality were robust and further supporting the reliability of our results. This is also consistent with the assumption proposed by Wen et al. that the COVID-19 pandemic had minimal impact on the associations between temperature variability and mortality (18).

This study systematically explored the impacts of three TV indices on mortality, with comprehensive stratified analyses by cause of death, gender, age group, and season, and further quantified the associated mortality burden using AF and AN. Yet several limitations of the present study should be acknowledged. First, as an ecological study, it only revealed the exposure-response relationship between temperature variations and mortality at the population level, and causal inference was limited due to the ecological fallacy. Second, population-level temperature data failed to reflect the actual individual exposure level, potentially leading to inaccurate exposure assessment and compromising the accuracy of association estimates. Third, given that the study was conducted in Chengdu, the provincial capital of Southwest China, the limited representativeness constrains the extrapolation of our findings to regions with substantially different climatic and demographic characteristics. Fourth, due to data availability, this study did not adjust for air pollutants and socioeconomic factors as potential confounders. Future studies should address these limitations to strengthen the robustness and generalizability of findings on the health effects of temperature variations.

## 5 Conclusion

In summary, this is the first study to systematically explore the associations between temperature variations and mortality in Chengdu, which not only investigated the impacts of total TV but also further decomposed it into intraday TV and interday TV to analyze their independent and differential effects on mortality risk. Our findings suggested that total TV exerts a significant adverse impact on mortality, with intraday TV identified as the primary driver of this health risk. Integrated health adaptation and intervention strategies should explicitly incorporate temperature variability as a core component, rather than focusing only on extreme weather events such as heatwaves and cold spells. Public health education should be strengthened to promote adaptive behaviors, and an early warning and forecasting system should be established for vulnerable populations, with the aim of reducing the mortality burden attributable to temperature variations. Future research can conduct multi-center validations in regions with diverse climatic and geographical features to further clarify the regional heterogeneity in the health impacts of temperature variations.

## Data Availability

The data analyzed in this study is subject to the following licenses/restrictions: Data were obtained from the Chengdu Meteorological Bureau and the Death Surveillance System of the Chinese Center for Disease Control and Prevention. The mortality dataset contains sensitive individual health information, is not publicly available due to ethical and privacy restrictions, and was used only for internal research purposes with official authorization. Requests to access these datasets should be directed to Chengdu Meteorological Bureau and the Death Surveillance System of China Center for Disease Control and Prevention.
