# Supplementary material for: Temperature variability and mortality risk: distinguishing intraday and interday effects and quantifying the attributable mortality burden in Chengdu, Southwest China
Source: Front Public Health. 2026 May 29;14:1809630. doi: 10.3389/fpubh.2026.1809630 (PMC13260626; doi:10.3389/fpubh.2026.1809630)

*Supplementary Material*

# Supplementary Tables

Table S1. Pearson correlation coefficients between the three TV indices and daily mean temperature at various lag intervals (lag 0-1 to 0-7 days).

| Variables | *Tmean* | *Total TV* | *Intraday TV* | *Interday TV* |
| --- | --- | --- | --- | --- |
| Lag 0-1 | | | | |
| *Tmean* | 1.000 |  |  |  |
| *Total TV* | 0.225 | 1.000 |  |  |
| *Intraday TV* | 0.233 | 0.996 | 1.000 |  |
| *Interday TV* | -0.023 | 0.174 | 0.124 | 1.000 |
| Lag 0-2 | | | | |
| *Tmean* | 1.000 |  |  |  |
| *Total TV* | 0.237 | 1.000 |  |  |
| *Intraday TV* | 0.252 | 0.992 | 1.000 |  |
| *Interday TV* | -0.027 | 0.231 | 0.139 | 1.000 |
| Lag 0-3 | | | | |
| *Tmean* | 1.000 |  |  |  |
| *Total TV* | 0.241 | 1.000 |  |  |
| *Intraday TV* | 0.263 | 0.987 | 1.000 |  |
| *Interday TV* | -0.036 | 0.283 | 0.156 | 1.000 |
| Lag 0-4 | | | | |
| *Tmean* | 1.000 |  |  |  |
| *Total TV* | 0.243 | 1.000 |  |  |
| *Intraday TV* | 0.272 | 0.982 | 1.000 |  |
| *Interday TV* | -0.048 | 0.327 | 0.173 | 1.000 |
| Lag 0-5 | | | | |
| *Tmean* | 1.000 |  |  |  |
| *Total TV* | 0.242 | 1.000 |  |  |
| *Intraday TV* | 0.279 | 0.978 | 1.000 |  |
| *Interday TV* | -0.057 | 0.366 | 0.192 | 1.000 |
| Lag 0-6 | | | | |
| *Tmean* | 1.000 |  |  |  |
| *Total TV* | 0.240 | 1.000 |  |  |
| *Intraday TV* | 0.283 | 0.974 | 1.000 |  |
| *Interday TV* | -0.062 | 0.399 | 0.208 | 1.000 |
| Lag 0-7 (Primary) | | | | |
| *Tmean* | 1.000 |  |  |  |
| *Total TV* | 0.237 | 1.000 |  |  |
| *Intraday TV* | 0.285 | 0.970 | 1.000 |  |
| *Interday TV* | -0.067 | 0.427 | 0.221 | 1.000 |

*Tmean* denotes daily mean temperature.(Primary) denotes the parameter setting used in the main analysis.

Table S2. Sensitivity analyses of lag intervals (lag 0-1 to 0-7 days) for the three TV indices: Percentage changes in all-cause mortality risk associated with each IQR increase.

| Lag intervals | *Total TV* | *Intraday TV* | *Interday TV* |
| --- | --- | --- | --- |
| Lag 0-1 | 1.0 (0.2, 1.9) | 1.2 (0.4, 2.1) | -0.4 (-1.0, 0.2) |
| Lag 0-2 | 1.1 (0.3, 1.9) | 1.5 (0.7, 2.4) | -0.7 (-1.3, -0.1) |
| Lag 0-3 | 1.2 (0.4, 2.1) | 1.8 (1.0, 2.7) | -0.9 (-1.5, -0.3) |
| Lag 0-4 | 1.4 (0.6, 2.2) | 2.2 (1.3, 3.1) | -1.1 (-1.7, -0.5) |
| Lag 0-5 | 1.6 (0.7, 2.4) | 2.6 (1.7, 3.5) | -1.3 (-1.9, -0.7) |
| Lag 0-6 | 1.6 (0.7, 2.4) | 3.0 (2.0, 3.9) | -1.5 (-2.1, -0.9) |
| Lag 0-7 (Primary) | 1.4 (0.5, 2.3) | 2.9 (2.0, 3.8) | -1.7 (-2.3, -1.1) |

Table S3. Sensitivity analyses of degrees of freedom (df from 3 to 6) for natural splines of daily mean temperature and relative humidity: Percentage changes in all-cause mortality risk associated with each IQR increase in the three TV indices.

| df | *Total TV* | *Intraday TV* | *Interday TV* |
| --- | --- | --- | --- |
| 3 | 1.4 (0.5, 2.3) | 2.9 (2.0, 3.9) | -1.8 (-2.4, -1.2) |
| 4 (Primary) | 1.4 (0.5, 2.3) | 2.9 (2.0, 3.8) | -1.7 (-2.3, -1.1) |
| 5 | 1.4 (0.5, 2.3) | 2.9 (2.0, 3.8) | -1.7 (-2.3, -1.1) |
| 6 | 1.4 (0.5, 2.2) | 2.8 (1.8, 3.7) | -1.5 (-2.2, -0.9) |

Table S4. Sensitivity analyses of maximum lag days (21-28 days) for daily mean temperature and relative humidity in the cross-basis function: Percentage changes in all-cause mortality risk associated with each IQR increase in the three TV indices.

| Maximum lag days | *Total TV* | *Intraday TV* | *Interday TV* |
| --- | --- | --- | --- |
| 21 (Primary) | 1.4 (0.5, 2.3) | 2.9 (2.0, 3.8) | -1.7 (-2.3, -1.1) |
| 22 | 1.3 (0.4, 2.2) | 2.9 (1.9, 3.8) | -1.7 (-2.3, -1.1) |
| 23 | 1.3 (0.4, 2.2) | 2.8 (1.9, 3.8) | -1.7 (-2.3, -1.1) |
| 24 | 1.3 (0.5, 2.3) | 2.9 (1.9, 3.8) | -1.7 (-2.3, -1.1) |
| 25 | 1.4 (0.5, 2.3) | 2.9 (2.0, 3.9) | -1.6 (-2.3, -1.0) |
| 26 | 1.4 (0.5, 2.3) | 3.0 (2.0, 3.9) | -1.6 (-2.2, -1.0) |
| 27 | 1.5 (0.6, 2.4) | 3.0 (2.0, 3.9) | -1.6 (-2.2, -1.0) |
| 28 | 1.5 (0.6, 2.4) | 3.0 (2.1, 4.0) | -1.6 (-2.3, -1.0) |

Table S5. Sensitivity analyses by excluding the COVID-19 peak period (from December 1, 2022 to February 28, 2023): Percentage changes in all-cause mortality risk associated with each IQR increase in the three TV indices.

| Exclusion of COVID-19 | *Total TV* | *Intraday TV* | *Interday TV* |
| --- | --- | --- | --- |
| Yes | 1.1 (0.4, 1.7) | 2.1 (1.3, 2.9) | -1.4 (-1.9, -0.8) |
| No (Primary) | 1.4 (0.5, 2.3) | 2.9 (2.0, 3.8) | -1.7 (-2.3, -1.1) |

# Supplementary Figure

Figure S1. Exposure-response association between total TV and all-cause mortality.


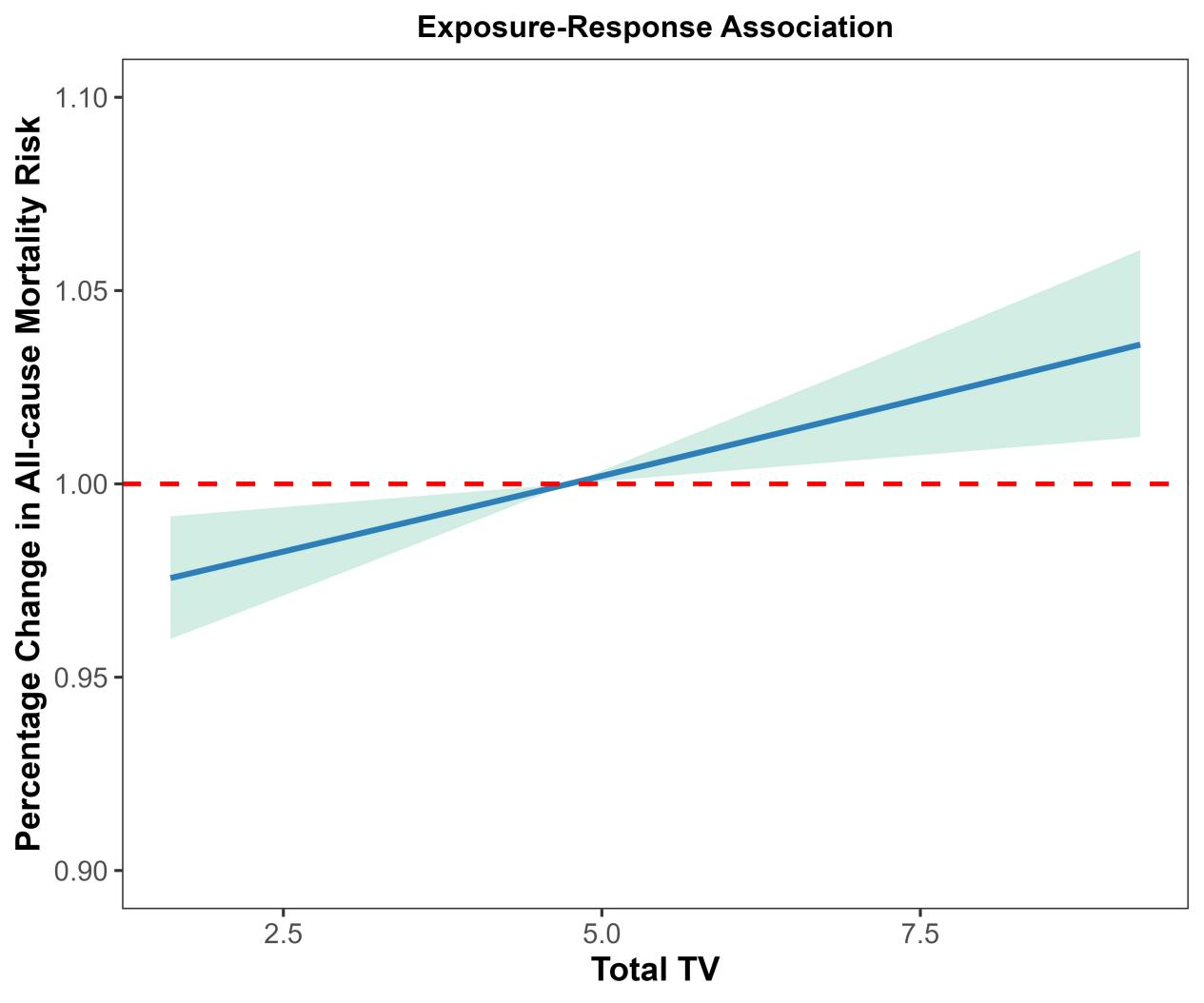

Supplement: Supplementary file 1 [file Data_sheet_1.docx]
